# Genome-Wide Identification of the Phosphoglycerate Kinases and Functional Analysis of *GmPGK5* in Regulating Oil Accumulation in Soybean

**DOI:** 10.3390/plants15111693

**Published:** 2026-05-30

**Authors:** Kai Zhang, Fengjia Zhu, Xiuli Yue, Songnan Yang, Yajun Mo, Shancen Zhao, Junyi Gai, Yan Li

**Affiliations:** 1 State Key Laboratory of Crop Genetics & Germplasm Enhancement and Utilization, National Center for Soybean Improvement, Key Laboratory for Biology and Genetic Improvement of Soybean, Jiangsu Collaborative Innovation Center for Modern Crop Production, Nanjing Agricultural University, Nanjing 210095, China; zkadrian@163.com (K.Z.);; 2 BGI Genomics, BGI-Shenzhen, Shenzhen 518083, China

**Keywords:** soybean, phosphoglycerate kinase, fatty acid, *GmPGK5*, hairy roots, dCAPS

## Abstract

Phosphoglycerate kinase (PGK) is a vital glycolytic enzyme that provides energy and carbon skeletons to support fatty acid synthesis. However, the *PGK* gene family has not been characterized in soybean (*Glycine max*), and its role in soybean oil accumulation remains unclear. Here, we identified six *GmPGK* genes in soybean, all of which encode proteins containing conserved PGK domains. Phylogenetic analysis clustered soybean PGK proteins into three groups. Analysis of *GmPGK* promoters revealed relatively abundant *cis*-elements related to plant growth, development, and phytohormone response. Expression profiling showed that *GmPGK5* transcript abundance increases progressively with oil accumulation during seed development, and is significantly higher in the high-oil variety NN1138-2. Overexpression of *GmPGK5* significantly increased total fatty acid content in soybean hairy roots. A single nucleotide polymorphism (SNP) located at Chr15:49447855 within the *GmPGK5* promoter was significantly associated with both seed oil content and seed weight in natural soybean accessions. Based on this SNP, a derived cleaved amplified polymorphic sequence (dCAPS) marker was developed to facilitate soybean molecular breeding. Our findings suggest that *GmPGK5* may positively regulate fatty acid accumulation in soybean. The identified natural variation and dCAPS marker provide potential valuable tools for marker-assisted selection to improve soybean oil content and seed weight.

## 1. Introduction

Soybean [*Glycine max* (L.) Merr.] supplies approximately 29% global vegetable oil and 69% dietary protein intake (http://www.soystats.com/), making it one of the most important oilseed crops. Soybean oil is composed mainly of five fatty acids (FAs), including linolenic acid (C18:3), linoleic acid (C18:2), oleic acid (C18:1), stearic acid (C18:0), and palmitic acid (C16:0) [1]. Of the total fatty acids, 60% are unsaturated, which provide beneficial effects on human health [2,3]. Therefore, improving the proportion of unsaturated fatty acids while increasing total fatty acid accumulation has been regarded as a key target for soybean improvement.

In plants, triacylglycerols (TAGs) serve as the primary form of oil storage [4]. Increasing FA content promotes the accumulation of both TAG and total oil content. Acetyl-CoA, the direct precursor for FA synthesis, is primarily derived from pyruvate generated by glycolysis. In addition, the ATP produced during glycolysis supplies the energy required for both FA synthesis and TAG assembly [5,6]. In sunflower and rapeseed seeds, the enzymes involved in glycolytic pathway exhibit increased activities during oil accumulation [7,8]. In addition, the activities of several key glycolytic enzymes are consistently higher in high-oil sunflower accessions than in low-oil sunflower accessions. Moreover, overexpression of the regulatory gene *BnLEC1* leads to the upregulation of multiple downstream glycolysis-related genes and an increase in seed oil content [9]. Therefore, the glycolytic pathway plays a pivotal role in determining oil content.

Most studies on soybean oil biosynthesis have focused on key enzymes or transcription factors involved in FA metabolism or TAG assembly. For instance, *FAD2* and *FAD3* encode fatty acid desaturases that influence the composition of unsaturated FAs [10,11], and *GmDGAT2A* catalyzes the final step of TAG synthesis [12]. Several transcription factors, including *GmDOF4*, *GmbZIP123*, *GmMYB73*, *GmZF392*, and *GmZF351* [13,14,15,16], have been found to regulate oil accumulation through modulating lipid synthesis genes. However, the roles of genes in upstream core metabolic pathways, particularly glycolysis, in modulating oil content remain largely unexplored.

As a key glycolytic enzyme, phosphoglycerate kinase (PGK) converts 1,3-bisphosphoglycerate (1,3-BPGA) and ADP to 3-phosphoglycerate (3-PGA) and ATP, thereby generating ATP in the glycolytic pathway [17,18]. Nearly all organisms ranging from bacteria to humans harbor PGK protein [19], which possesses a highly conserved amino acid sequence [20]. The tertiary structure of PGK comprises two distinct domains. The N-terminal domain recognizes and binds either 3-PGA or 1,3-BPGA, whereas the C-terminal domain interacts with ADP or ATP [21]. In wheat, two highly conserved PGK isoforms have been identified [20], one primarily localized in the cytoplasm and the other in plastid [22]. The *PGK* gene family performs diverse functions in plants. In *Arabidopsis thaliana*, three *AtPGK* gene members were detected [23]. *AtPGK1* is specifically expressed in chloroplasts; *AtPGK2* in chloroplasts and plastids, while *AtPGK3* in the cytoplasm. Four *PGK* members were identified in rice, and the allele of *OsPgk2a-*P was salt-inducible and improved both salt tolerance and yield when expressed in transgenic tobacco [24]. To date, there is no report on comprehensive identification and analysis of soybean *PGK* gene family, nor their involvement in soybean oil accumulation.

In this study, we systematically characterized the soybean *GmPGK* gene family, including their phylogenetic relationships, protein conserved motifs and structures, promoter *cis*-regulatory elements, and transcriptional expression patterns. Furthermore, we validated the function of *GmPGK5* by overexpressing it in soybean hairy roots and examined its natural allelic variations in two diverse panels of soybean accessions. Our results indicate that *GmPGK5* is associated with fatty acid content, and provide a novel molecular marker for breeding high-oil, large-seed soybean varieties.

## 2. Results

### 2.1. Identification and Phylogenetic Characterization of GmPGK Family Members

To identify candidate soybean PGK family members, we performed Hidden Markov Model (HMM) screening using the PGK family profile (PF00162). A total of six PGK family members were identified in soybean and designated GmPGK1–GmPGK6. These GmPGKs proteins vary in sequence length, spanning from 401 to 674 amino acids. Their molecular weights and predicted isoelectric points range from 42,391.94 Da to 76,232.69 Da and from 5.96 to 8.11, respectively (Table S1).

**Figure 1:**
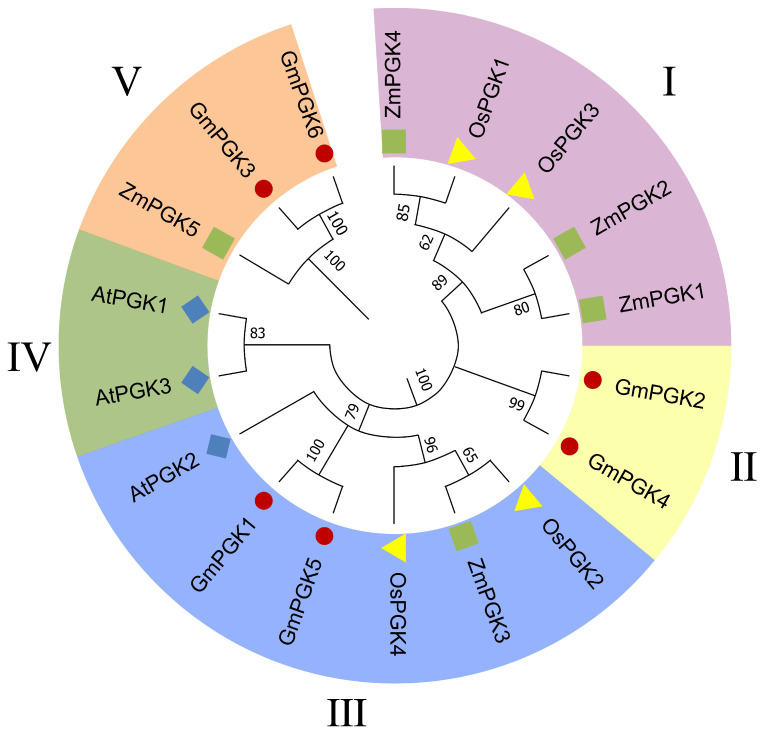
Phylogenetic tree of PGK family. Soybean (*Glycine max*), maize (*Zea mays*), rice (*Oryza sativa*), and *Arabidopsis thaliana* are indicated by Gm, Zm, Os, and At, respectively. The phylogenetic tree was generated using the Maximum Likelihood (ML) method based on the amino acid sequences of PGK proteins. Bootstrap values from 1000 replicates are shown at nodes. I–V represent five groups, each with a different colored background.

A phylogenetic tree was generated using the protein sequences of six soybean GmPGK members, three *Arabidopsis* AtPGK members, four rice OsPGK members, and five maize ZmPGK members (Figure 1). Gene IDs for all *PGK* genes are listed in Table S2. Phylogenetic analysis clustered these PGK proteins into five groups, with Group III containing the most members while Groups II and IV the fewest. Among the four species, soybean possessed the most PGK members (six, distributed in Group II, III and V), whereas *Arabidopsis* harbored the fewest (three).

### 2.2. Motifs, Conserved Domains and Structures of GmPGK Proteins

**Figure 2:**
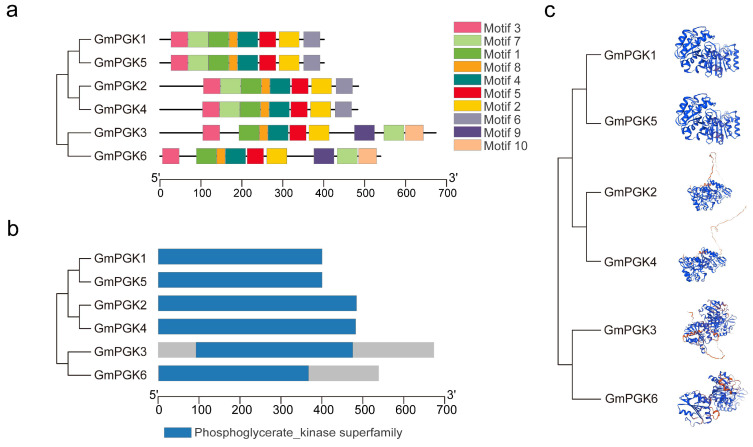
Motifs, domains and predicted structures of GmPGK proteins. (**a**) Conserved motifs 1–10 are shown in distinct colors. (**b**) The phosphoglycerate kinase (PGK) domain and non-conserved regions are represented by blue and gray boxes, respectively. (**c**) Predicted three-dimensional structures of GmPGK proteins using SWISS-MODEL web-based service with AlphaFold2 model.

To further characterize GmPGK proteins, their motifs, conserved domains and structures were investigated (Figure 2). All GmPGKs contain the conserved motifs 1, 2, 3, 4, 5, and 8 (Figure 2a), suggesting these motifs are likely essential for their functions. The positions and numbers of these motifs vary across different phylogenetic groups; however, members within the same group share identical motif compositions. For example, GmPGK1 and GmPGK5, GmPGK2 and GmPGK4, and GmPGK3 and GmPGK6 exhibit the same motif sets. Furthermore, although the positions and lengths of conserved domains differ between groups, they were generally similar within the same group (Figure 2b). Moreover, within the same group, GmPGK proteins exhibited similar predicted subcellular localizations (Table S1). For example, GmPGK1 and GmPGK5 were predicted to be localized in the cytoplasm, while GmPGK2 and GmPGK 4 were predicted to be in the chloroplast. Consistently, GmPGKs within the same group also share similar predicted three-dimensional protein structures (Figure 2c), which further supports that members within the same group likely possess similar biological functions.

### 2.3. Promoter Cis-Acting Elements of GmPGK Genes

**Figure 3:**
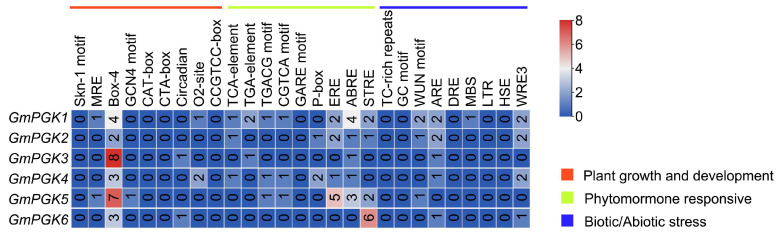
*Cis*-acting elements in the promoter sequences of *GmPGK* genes. Numbers in boxes represent the element counts in corresponding genes.

The *cis*-acting elements within the promoter regions of *GmPGK* genes were grouped into three categories (Figure 3). The first category is related to plant growth and development, including Skn-1 motif, MRE, Box-4, GCN4 motif, CAT-box, CTA-box, Circadian and O2-site. The second category corresponds to phytohormone response, including nine elements, such as the CGTCA motif, TGA-element, TGACG motif, ERE and STRE elements. The third category features biotic/abiotic stress-related elements, which comprise nine members: TC-rich repeats, GC motif, WUN motif, and others [25]. All *GmPGK*s contain the Box-4 element, and elements in the first and second categories were relatively abundant. Notably, *GmPGK5* contains seven Box-4 and five ERE elements. Taken together, these findings indicate the involvement of soybean *PGK* genes in plant growth, development, hormone signal transduction, and stress response.

### 2.4. Expression Patterns of GmPGK Genes

**Figure 4:**
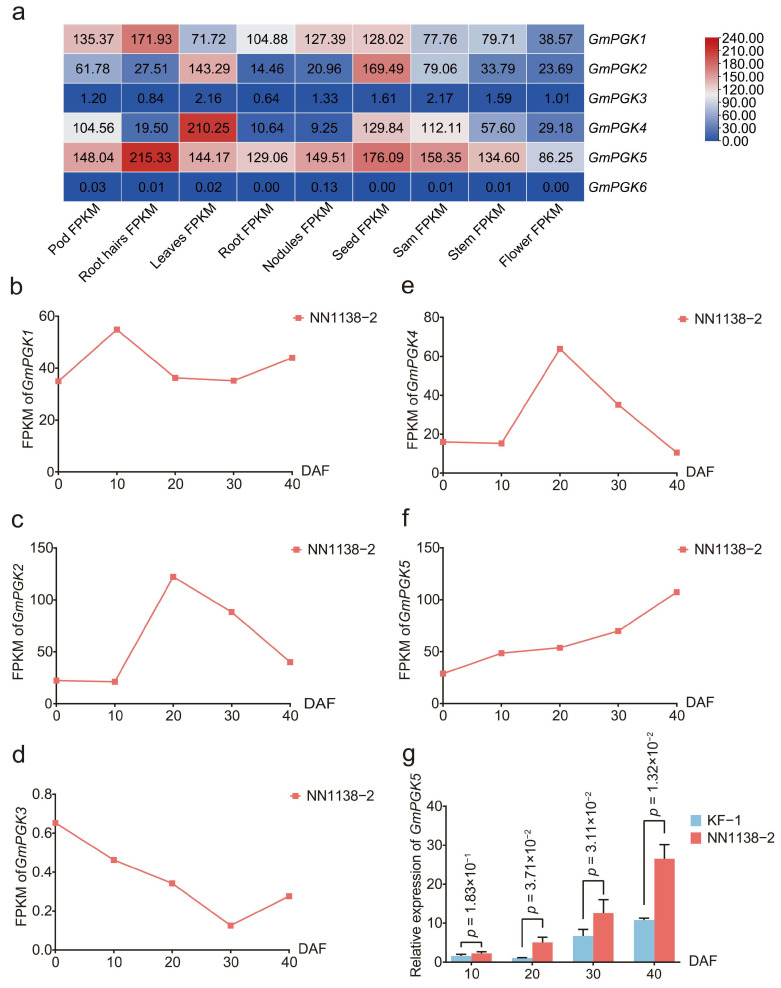
Expression profiles of *GmPGK* genes. (**a**) Heatmap of *GmPGK* expression profiles in soybean tissues. Data are Fragments Per Kilobase per Million mapped reads (FPKM) from Phytozome database. (**b**–**f**) Expression patterns of *GmPGK*s in developing seeds of NN1138-2 at 0, 10, 20, 30, and 40 days after flowering (DAF). FPKM values were downloaded from BioProject (accession: PRJNA539842). (**g**) Reverse transcription quantitative PCR (RT-qPCR) analysis of *GmPGK5* relative expression in KF-1 and NN1138-2 seeds at 10, 20, 30, and 40 DAF. Expression levels were normalized to the reference gene *GmUKN1* and calibrated to KF-1 at 20 DAF. Values shown are means ± SD from three biological replicates. Two tailed Student’s *t*-tests were used for statistical comparison between KF-1 and NN1138-2.

To characterize the transcriptional expression patterns of *GmPGK* genes, we retrieved their expression profiles in soybean different soybean tissues from Phytozome (Table S3, Figure 4a). Among the six *GmPGK* genes, *GmPGK6* displayed extremely low expression (FPKM < 0.2) in all tissues, suggesting that it may be a pseudogene or have a highly restricted expression pattern. *GmPGK3* also showed consistently low transcript levels (FPKM < 3) in most tissues. The remaining four *GmPGK* genes were expressed in all tissues examined (FPKM > 10). Specifically, *GmPGK2* and *GmPGK4* displayed varying expression levels, with particularly elevated abundance in leaves and seeds; whereas *GmPGK1* and *GmPGK5* exhibited relatively high expression levels across all tissues.

To explore the roles of *GmPGK* genes during soybean seed development, the expression profiles of *GmPGK* genes in developing seeds (Table S4) were obtained from transcriptomic data of the soybean variety NN1138-2 (NCBI SRA accession number PRJNA539842). Consistent with the previous report that oil content increases as seed development progresses [26], expression analysis of *GmPGK* genes in developing soybean seeds (Figure 4b–f) revealed that only *GmPGK5* exhibited a continuous increase in transcript abundance (Figure 4f), paralleling the oil accumulation pattern. In addition, reverse transcription quantitative PCR (RT-qPCR) with gene-specific primers (Table S5) showed that *GmPGK5* expression was higher in the high-oil variety NN1138-2 at 20, 30, and 40 days after flowering (DAF) (Figure 4g), which corresponded to the oil content phenotype. These results suggest that *GmPGK5* is likely involved in regulating soybean oil content.

### 2.5. Effect of GmPGK5 Overexpression on Oil Content

**Figure 5:**
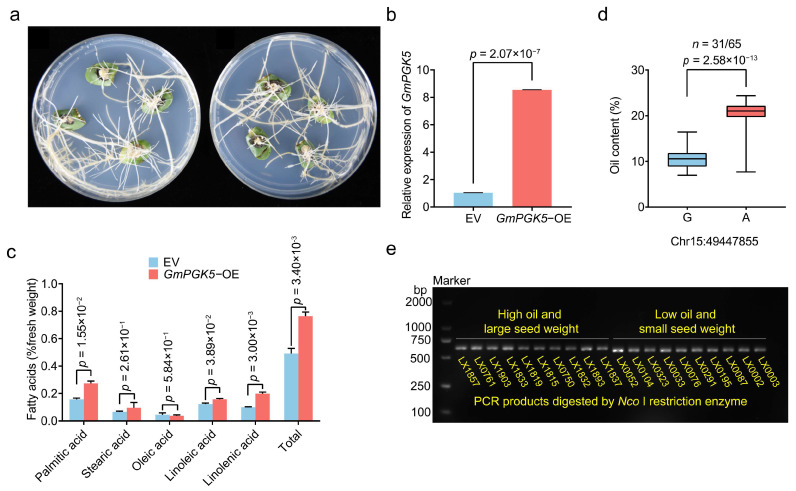
Effect of *GmPGK5* overexpression and allelic variation on oil content. (**a**) Transgenic soybean hairy roots carrying the empty vector (EV, left) or overexpressing *GmPGK5* (*GmPGK5*-OE, right). (**b**) *GmPGK5* relative expression in transgenic soybean hairy roots, normalized to the reference gene *GmUKN1*. Data are mean ± SD with three biological replicates. (**c**) Fatty acids contents in transgenic soybean hairy roots. In (**b**,**c**), two tailed Student’s *t*-tests were used to determine statistical significances. (**d**) Allelic effect of the SNP (Chr15:49447855) in *GmPGK5* on seed oil content in 96 soybean accessions. Statistical comparison was performed via two-sided *Wilcoxon* test. (**e**) The dCAPS marker designed for the SNP (Chr15:49447855) in *GmPGK5*. Representative gel electrophoresis image of the dCAPS marker genotyped in 20 extreme soybean accessions exhibiting contrasting oil content and 100-seed weight phenotypes (Table S8).

As shown in Figure 5a,b, overexpressing *GmPGK5* in soybean hairy roots significantly increased *GmPGK5* transcript levels compared to controls. Fatty acid analysis further revealed that *GmPGK5*-overexpressing roots accumulated significantly more total fatty acids, as well as higher content of palmitic acid, linoleic acid, and linolenic acid (Figure 5c). Thus, *GmPGK5* positively modulates soybean oil content by promoting fatty acid accumulation in hairy roots.

### 2.6. Sequence Variations in GmPGK5 and Their Allelic Effects on Soybean Seed Oil Content

The coding sequence of *GmPGK5* in KF-1 is identical to that of NN1138-2, while 25 sequence variations were identified in *GmPGK5* promoter region, of which four were insertions/deletions (InDels) and the remaining 21 were SNPs (Table S6). These sequence variations suggest that the differential expression of *GmPGK5* between the two varieties (Figure 4g) might be caused by the polymorphisms in its promoter region, which could contribute to differences in seed oil content.

To determine whether different allelic variants of *GmPGK5* in KF-1 and NN1138-2 were associated with oil content, we analyzed their allelic effects on seed oil content in 96 soybean accessions. Among the 25 sequence variations in the promoter of *GmPGK5*, 21 SNPs had a minor allele frequency (MAF) of 0.05 or higher (Table S7). The SNP at Chr15:49447855 showed the strongest association (lowest *p* value, Table S7) with seed oil content in these 96 accessions (Figure 5d), with the A allele conferring significantly higher seed oil content than the G allele (Figure 5d). In addition, this SNP also showed a significant association with seed oil content and 100-seed weight in 19,914 accessions of the USDA soybean germplasm collection from SoyGVD database (Figure S1). Accordingly, a dCAPS marker was developed for this SNP. The PCR product of the G genotype was cleaved by the restriction enzyme *Nco* I, producing a 578 bp fragment, whereas the A genotype PCR product remained undigested, yielding a 604 bp fragment. Then, we selected 40 soybean accessions with extreme phenotypes in soybean seed oil content and 100-seed weight to verify the accuracy of this marker (Table S8). Gel electrophoresis analysis showed that all 20 low-oil and small-seed accessions carried the G genotype, while all 20 high-oil and large-seed accessions carried the A genotype, effectively (100% concordance between genotype and phenotype in these extreme lines) distinguishing the two genotypes (Figure 5e and Figure S2). This natural sequence variation in *GmPGK5* exhibits significant association with oil content and seed weight, offering a potential marker for marker-assisted breeding.

### 2.7. Prediction of GmPGK5-Interacting Proteins

**Figure 6:**
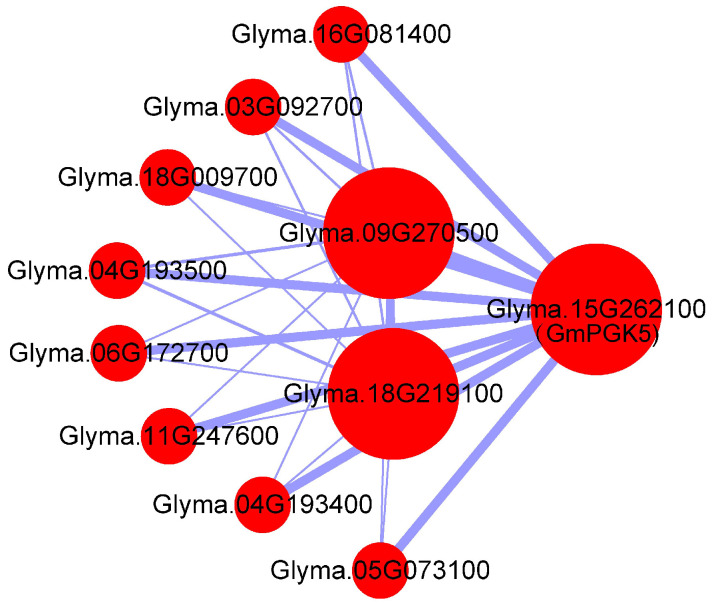
Predicted interacting proteins of GmPGK5 using STRING database. Circle size represents the number of interacting proteins and line thickness indicates interaction strength.

Ten proteins potentially interacting with GmPGK5 were predicted using the STRING database (Figure 6). Subsequently, we carried out Gene Ontology (GO) analysis of these interacting proteins, and the GO annotations are listed in Table S9. The predicted interacting proteins were involved in glucose metabolic process and glycolysis, including glyceraldehyde-3-phosphate dehydrogenase, which functions adjacent to PGK in the glycolytic pathway [27,28].

## 3. Discussion

The *PGK* encodes an important enzyme in glycolysis and regulates multiple aspects of plant growth and development. Studies in *Arabidopsis* and rice have revealed that several *PGK* homologs modulate development and salt tolerance [23,24]. However, the *PGK* family in soybean remains uncharacterized, and its contribution to seed oil content is unknown. Here, we identified six *PGK* gene family members in soybean. Two of them, *GmPGK1* and *GmPGK5*, cluster with *AtPGK2* (Figure 1), suggesting a potential function similar to *AtPGK2*. Additionally, multiple *cis*-acting elements in *GmPGK* promoters were related to plant growth, development, and hormone responses. Notably, *GmPGK5* contained seven Box-4, five ERE, and two STRE elements in its promoter (Figure 3). ERE and STRE elements participate in plant development and hormonal regulation [29,30], implying that *GmPGK5* may play significant roles in these processes. However, the presence of *cis*-elements only provides circumstantial evidence, and the high copy numbers of Box-4 and ERE elements in the *GmPGK5* promoter raise the question of whether they function redundantly or synergistically. To dissect their individual contributions, promoter deletion analyses and promoter:GUS reporter assays will be needed in future work.

Several genes participating in oil synthesis have been discovered in soybean [3,10,15,16,31,32]. Moreover, previous studies in sunflower and rapeseed have shown that overexpression of glycolytic enzyme genes results in enhanced oil accumulation [7,8]. However, the function of *GmPGK*s in soybean oil accumulation remains unexplored. To further characterize the biological functions of *GmPGK*s in soybean, we examined their expression profiles (Figure 4). Only *GmPGK5* exhibited an expression pattern (Figure 4b–f) that paralleled oil accumulation during seed development [3,26,33]. Additionally, expression level of *GmPGK5* in seeds after 20 DAF was significantly higher in high-oil variety NN1138-2 (Figure 4g). These findings suggest that *GmPGK5* may act as a pivotal regulator of oil accumulation in soybean. This hypothesis is supported by the increased fatty acid content in *GmPGK5*-overexpressing soybean hairy roots, demonstrating that *GmPGK5* positively regulates fatty acid accumulation in this system. The increase in polyunsaturated fatty acids may result from enhanced substrate availability or indirect activation of downstream desaturases such as *FAD2* and *FAD3*. It should be noted, however, that while transgenic soybean hairy roots provide a rapid system for investigating gene function, fatty acid synthesis in roots may differ from that in developing seeds. Therefore, further validation using stable transformation with a native or seed-specific promoter is needed to confirm the biological function of *GmPGK5* during seed development in future studies.

In addition, allelic effect analysis revealed that the SNP at Chr15:49447855 within the *GmPGK5* promoter had a significant effect on seed oil content in 96 soybean accessions (Figure 5d). Notably, this SNP was also significantly associated with seed oil content and seed weight in a larger population of the USDA soybean germplasm collection in the SoyGVD database. A corresponding dCAPS marker was successfully developed to distinguish the two alleles of this SNP, and validated in 20 extreme accessions with contrasting oil content and seed weight, thereby enabling its potential application as a promising tool for marker-assisted selection in breeding high-oil and large-seed soybean cultivars.

Predictions of *GmPGK5*-interacting proteins indicate that GmPGK5 may interact with proteins involved in glycolysis, particularly glyceraldehyde-3-phosphate dehydrogenase (GAPDH), which functions closely with PGK in the glycolytic pathway. This is supported by previous reports that *GmGAPDH* overexpression increases oil content in seeds [34,35], providing indirect evidence for a role of *GmPGK5* in lipid metabolism. Nevertheless, STRING predictions rely primarily on co-expression and text mining rather than direct biochemical evidence. Therefore, these interactions should be considered as hypotheses requiring future experimental validation, such as yeast two-hybrid or co-immunoprecipitation assays. Furthermore, the precise mechanism by which *GmPGK5* overexpression increases fatty acid levels remains unclear. Theoretically, enhanced PGK activity could boost ATP production and thereby promote fatty acid synthesis—a hypothesis that awaits direct biochemical testing. Alternatively, an indirect effect, possibly mediated by altered expression of other oil biosynthesis-associated genes not examined here, may also contribute. All these questions will need to be addressed in future studies.

## 4. Materials and Methods

### 4.1. Identification and Characterization of GmPGK Genes

We acquired genomic and protein sequences of the *GmPGK* genes from Phytozome database (https://phytozome-next.jgi.doe.gov/) using the soybean reference genome (Wm82.a2.v1) [36]. Soybean PGK proteins were screened using a simple HMM search in TBtools (v2.210) with the PGK domain (PF00162) retrieved from the Pfam database (https://www.ebi.ac.uk/interpro/entry/pfam/#table, accessed on 5 August 2023) [37], with E-value threshold set to 1 × 10^−5^. In parallel, a BLASTP search was executed using known Arabidopsis AtPGK protein sequences as queries [23,38]. Candidates identified by both methods were further validated by confirming the presence of PGK conserved domain through the CDD database (https://www.ncbi.nlm.nih.gov/Structure/cdd/cdd.shtml, accessed on 5 August 2023) [39]. The ProtParam at ExPASy server (https://web.expasy.org/protparam, accessed on 23 December 2023) [40] was employed to predict the molecular weights and isoelectric points of GmPGKs (Table S1). The subcellular localization of GmPGK proteins were predicted using WoLF PSORT (https://wolfpsort.hgc.jp/) [41].

### 4.2. Characterization of Conserved Motifs, Protein Structures and Phylogenetic Relationship

Conserved motifs were identified from all GmPGK protein sequences using MEME (v5.5.7, default parameters) at http://meme-suite.org/tools/meme (accessed on 23 December 2023) [42]. Motif diagrams were generated using TBtools [37]. The amino acid sequences of PGKs (Table S2) from *Arabidopsis thaliana* (TAIR11), rice (MSU v7.0), maize (var. B73), and soybean (W82.a2.v1) were downloaded from Phytozome database (https://phytozome-next.jgi.doe.gov/), then subjected to protein structure prediction using SWISS-MODEL (https://swissmodel.expasy.org/interactive, accessed on 21 May 2026) with AlphaFold2 [43], sequence alignment, and phylogenetic analysis in MEGA 11.0.13 (https://www.megasoftware.net/older_versions, accessed on 9 August 2026). Multiple sequence alignment was performed using ClustalW in MEGA 11.0.13 with the default parameter settings: for multiple alignment, the gap opening penalty was set to 10.00 and the gap extension penalty to 0.20, and delay divergent cutoff (%) was set to 30. The phylogenetic tree was constructed using the Maximum Likelihood (ML) method with 1000 bootstrap replicates. The Jones–Taylor–Thornton (JTT) model was selected as the amino acid substitution model. All sites were used for gap/missing data treatment. The ML heuristic method was set to Nearest-Neighbor-Interchange (NNI), with the initial tree generated automatically (BioNJ) and no branch swap filter applied [44].

### 4.3. Cis-Acting Elements Analysis of GmPGK Promoters

The *cis*-acting elements in the 2000 bp sequences upstream of the ATG start codon for all *GmPGK* genes were identified in the PlantCARE database (https://bioinformatics.psb.ugent.be/webtools/plantcare/html/, accessed on 19 May 2025) [45], and were then classified and counted. Heatmap was generated using TBtools (v2.210) [37].

### 4.4. Plant Materials, Growth Conditions, and Sample Collection

Soybean accessions were planted at the Dangtu Experimental Station (Maanshan, China) in June 2019, and seed samples were collected at 10, 20, 30, and 40 DAF.

### 4.5. Expression Profiles of GmPGK Genes

To dissect the expression patterns of *GmPGK* genes, their FPKM values from the Phytozome database and BioProject (accession: PRJNA539842) [26] were retrieved, and then used to generate a heatmap using TBtools (v2.210) [37] and for expression pattern analysis during seed development.

### 4.6. RNA Isolation and RT-qPCR Analysis

Total RNA was isolated from soybean tissues using the RNAprep Pure Plant Plus Kit (Tiangen, Beijing, China), and reverse-transcribed into cDNA with the HiScript II Q RT Super Mix for qPCR kit (R223-01, Vazyme, Nanjing, China). RT-qPCR primers (listed in Table S5) were designed via Primer-BLAST (https://blast.ncbi.nlm.nih.gov/Blast.cgi, accessed on 5 December 2023). The RT-qPCR experiment was carried out on LightCycler 480 Real-Time Detection System (Roche Diagnostics Ltd., Rotkreuz, Switzerland) using ChamQ Universal SYBR qPCR Master Mix kit (Q711-02, Novizan, Nanjing, China). Three biological replicates were analyzed per sample [46]. The 2^−∆∆Ct^ calculation approach was applied to quantify relative expression levels [47].

### 4.7. Genetic Transformation and Fatty Acid Quantification

We inserted the coding sequence of *GmPGK5* into pBinGFP4 vector after the CaMV 35S promoter. Then the empty vector and 35S:*GmPGK5* construct were separately transformed into *Agrobacterium tumefaciens* strain K599, which was then inoculated onto soybean cotyledonary nodes on 1/2 MS medium to generate transgenic soybean hairy roots [48]. After 30 days, positive transgenic soybean hairy roots were identified by green fluorescence under a macro fluorescence microscope (MVX10, Olympus Corporation, Tokyo, Japan), carefully blotted dry, and 0.100 g of fresh roots was weighed. Root samples were placed in grinding tubes containing 1 mL of extraction solution (2.5% H_2_SO_4_ in methanol, *v*/*v*) and 20 µL of an internal standard (0.1 g of methyl heptadecanoate dissolved in 10 mL of ethyl acetate). After incubation at 85 °C for 1 h, the samples were centrifuged at 3381× *g* for 5 min. A total of 500 µL of the resulting supernatant was transferred into a fresh tube and mixed with 600 µL of 0.9% NaCl and 350 µL of chromatography-grade n-hexane. The mixture was then vortexed for 1 min, followed by another centrifugation step at 1503× *g* for 10 min. Then 200 µL of the supernatant was transferred to a liquid chromatography vial for fatty acid analysis using a gas chromatograph (ThermoScientific Trace GC Ultra, Waltham, MA, USA) [3].

### 4.8. Allelic Variation Analyses and Derived Cleaved Amplified Polymorphic Sequence Marker Development for GmPGK5

The promoter and coding sequences of *GmPGK5*, were cloned from soybean accessions of KF-1 and NN1138-2 (Table S6), and sequenced at Tsingke Biotech Co., Ltd. (Beijing, China). Allelic effects were analyzed using the SNPs (MAF ≥ 0.05) in *GmPGK5* from the resequencing data generated by BGI (https://www.genomics.cn/) [49] and seed oil content [3] of 96 soybean accessions (Table S7). Association of the SNP at Chr15:49447855 with soybean seed weight and oil content was also analyzed in SoyGVD database (https://yanglab.hzau.edu.cn/SoyGVD/#/, accessed on 21 May 2026).

The dCAPS marker was developed based on the SNP at Chr15:49447855 in *GmPGK5* promoter region, and tested among 40 soybean accessions with extreme seed oil content and 100-seed weight (Table S8). We used dCAPS Finder 2.0 to analyze if the SNP was located at a restriction enzyme recognition site, allowing the identification of the most suitable enzyme cleavage site for marker design [50]. Then, the primer (Table S5) was designed via Primer Premier 5 software. The amplification products were digested with *Nco* I restriction enzyme. The digestion reaction system was as follows: 5 µL of amplified product, 1 µL of Buffer, 0.2 µL of *Nco* I enzyme, and 3.8 µL of ddH_2_O. The reaction mixture was incubated at 37 °C for 2 h to allow digestion. Subsequently, the digested products were electrophoresed on a 2% agarose gel for 90 min, and the results were recorded using a Gel Imaging System (Bio-Rad, Hercules, CA, USA).

### 4.9. Prediction of Interacting Proteins

Potential proteins interacting with GmPGK5 were predicted via the STRING database (https://cn.string-db.org/, accessed on 12 February 2026), with the combined score filtered at 0.9 or higher. The protein interactions were then visualized by Cytoscape (v3.7.1). GO annotation of GmPGK5 interacting proteins (Table S9) was retrieved from the SoyBase database (https://www.soybase.org/, accessed on 12 February 2026).

### 4.10. Statistical Analysis

All experiments were carried out with three independent replicates and three individual plants. Normality of the data was assessed using the D’Agostino–Pearson test in Graphpad Prism 8, and homogeneity of variances was evaluated using the F test. For comparisons between two groups, two-tailed Student’s *t*-tests were applied when the assumptions of normality and equal variance were met; otherwise, the two-sided *Wilcoxon* tests were used.

## 5. Conclusions

In summary, we present a systematic characterization of the soybean *GmPGK* gene family, and reveal that *GmPGK5* is likely to play a role in soybean fatty acid accumulation. Analyses of transgenic hairy roots and natural variations indicate that *GmPGK5* affects both oil content and seed weight in soybean. Additionally, the dCAPS marker developed here has application potential for marker-assisted selection in breeding high-oil and large-seed soybean cultivars. Collectively, these findings may serve as a reference for future research into the molecular basis of fatty acid accumulation in soybean.

## Data Availability

The original contributions presented in this study are included in the article/Supplementary Materials. Further inquiries can be directed to the corresponding author.

## Supplementary Materials

The following supporting information can be downloaded at https://www.mdpi.com/article/10.3390/plants15111693/s1, Figure S1. Box plots of the allelic effect of the SNP at Chr15:49447855 in *GmPGK5* on soybean oil content and 100-seed weight. Figure S2. Validation of the dCAPS marker for the SNP (Chr15:49447855) in *GmPGK5* using 20 additional soybean accessions (Table S8) with extreme phenotypes for seed oil content and 100-seed weight. Table S1. Characterization of PGK proteins in Soybean. Table S2. Gene names and IDs of all *PGK* genes in this study. Table S3. FPKM values of *GmPGK*s in soybean tissues from Phytozome database. Table S4. FPKM values of *GmPGK*s in soybean seeds at 0, 10, 20, 30, and 40 DAF in NN1138-2. Table S5. Primers used in this study. Table S6. Sequence variations in the promoter region of *GmPGK5* between KF-1 and NN1138-2. Table S7. Allelic variations in *GmPGK5* and seed oil content of 96 soybean accessions. Table S8. The 100-seed weight and seed oil content of 40 extreme soybean accessions. Table S9. Gene annotation and GO analysis of predicted GmPGK5-interacting proteins.

## Abbreviations

The following abbreviations are used in this manuscript:

DAF	Days after flowering
dCAPS	Derived cleaved amplified polymorphism sequences
FA	Fatty acid
FPKM	Fragments per kilobase per million mapped reads
GO	Gene ontology
HMM	Hidden Markov model
InDel	Insertion/Deletion
ML	Maximum likelihood
PGK	Phosphoglycerate kinase
RT-qPCR	Reverse transcription quantitative PCR
SNP	Single nucleotide polymorphism
TAG	Triacylglycerol
