# Supplementary material for: Genome-Wide Identification of the Phosphoglycerate Kinases and Functional Analysis of GmPGK5 in Regulating Oil Accumulation in Soybean
Source: Plants (Basel). 2026 May 30;15(11):1693. doi: 10.3390/plants15111693 (PMC13259330; doi:10.3390/plants15111693)
Supplement: Supplementary file 1 [file plants-15-01693-s001.zip › Supplementary Figures 20260530.pdf]

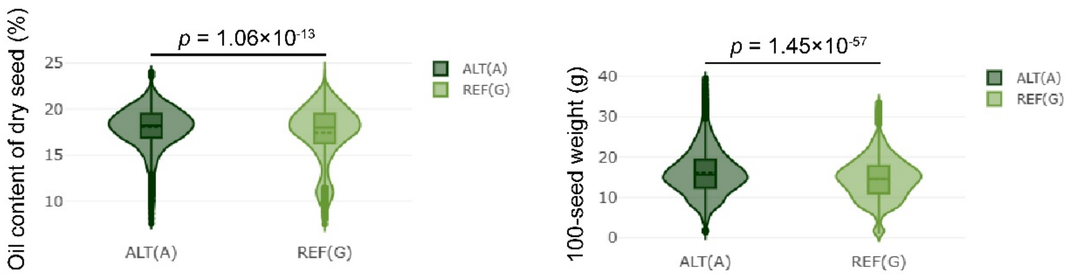

Figure S1. Box plots of the allelic effect of the SNP at Chr15:49447855 in *GmPGK5* on soybean oil content and 100-seed weight. A total of 19,914 soybean accessions from the SoyGVD database (https://yanglab.hzau.edu.cn/SoyGVD/#/) were analyzed, including 10,828 carrying genotype A and 9,086 carrying genotype G. Statistical significance was determined using the *Wilcoxon* test.

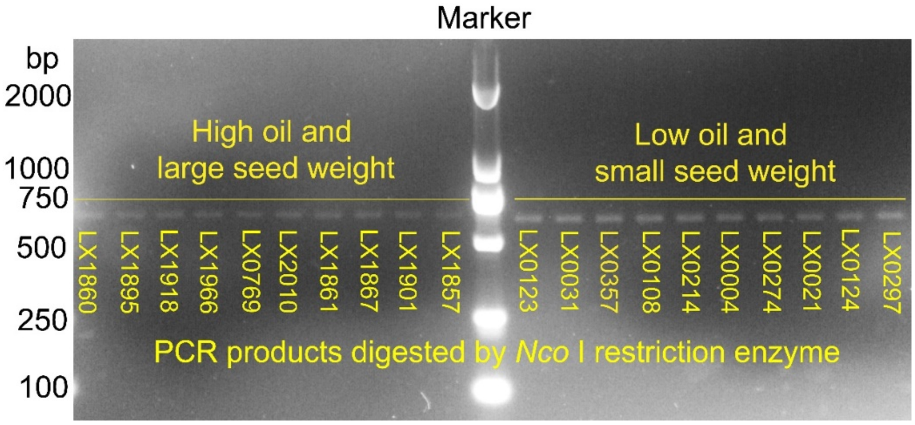

Figure S2. Validation of the dCAPS marker for the SNP (Chr15:49447855) in *GmPGK5* using 20 additional soybean accessions (Table S8) with extreme phenotypes for seed oil content and 100-seed weight.
